# The first mitochondrial genome of *Fistulobalanus albicostatus* (Crustacea: Maxillopoda: Sessilia) and phylogenetic consideration within the superfamily Balanoidea

**DOI:** 10.1080/23802359.2020.1788459

**Published:** 2020-07-11

**Authors:** Jun Song, Panpan Chen, Mei Tian, Nanjing Ji, Yuefeng Cai, Xin Shen

**Affiliations:** a Jiangsu Institute of Marine Resources/Jiangsu Key Laboratory of Marine Biotechnology, Jiangsu Ocean University, Lianyungang, China; b Guangdong Provincial Key Laboratory of Marine Biotechnology, Shantou University, Shantou, China; c Co-Innovation Center of Jiangsu Marine Bio-industry Technology, Jiangsu Ocean University, Lianyungang, China

**Keywords:** Sessilia, *Fistulobalanus albicostatus*, mitochondrial genome, phylogeny, non-monophyly

## Abstract

The first complete mitochondrial genome of the intertidal barnacle *Fistulobalanus albicostatus* Pilsbry, 1916 (Crustacea: Maxillopoda: Sessilia) is presented. The genome is a circular molecule of 15,665 bp, which encodes a set of 37 typical metazoan genes. All non-coding regions are 438 bp in length, with the longest one speculated as the control region (264 bp), which is located between *srRNA* and *trnI*. All protein-coding genes (PCGs) have an ATD (ATA, ATT, or ATG) start codon, except *nad1*, which is initiated with GTG. Remarkably, *cox3*, *cob*, *nad1-5* have incomplete stop codons (T–– or TA–) and the remaining PCGs have the complete stop codon (TAA). Phylogenetic analysis based on 13 mitochondrial PCGs shows that the members of the Archaeobalanidae and Balanidae intermingle with species from Pyrgomatidae. The results supposed that Balanidae and Archaeobalanidae are non-monophyly.

*Fistulobalanus albicostatus* Pilsbry, 1916 (Crustacea: Maxillopoda: Sessilia) is a common acorn barnacle in mangroves and on soft shores, inhabiting on rocks, mollusk shells, tree trunks, and mangrove leaves (Prabowo and Yamaguchi 2005; Chang et al. 2017; Hayashi 2017). The species is widely distributed in East Asia, including Japan, Korea, and China (Newman and Ross 1976; Chan and Leung 2007; Ping-Hung et al. 2007). The work presents the first complete mitochondrial genome of *F. albicostatus*. Specimen of *F. albicostatus* was collected from Qidong (32°1′24″N, 121°44′21″E), Jiangsu Province, China. Genomic DNA was isolated using TIANamp Marine Animal DNA Kit (TIANGEN, Beijing, China) following the manufacturer’s protocol, which was stored at Marine Museum of Jiangsu Ocean University (accession number: Fal-002). The amplification of internal fragments and long fragments followed the procedures described in our previous study (Chen et al. 2019). PCR products were purified (GeneMark), cloned (pGEMT easy, Promega, Madison, WI) and sequenced (MAP BIOTECH, Shanghai, China).

The complete mitochondrial genome of *F. albicostatus* is a circular molecule of 15,665 bp in length, encodes 13 protein-coding genes (PCGs), 2 ribosomal RNAs (rRNAs), 22 transfer RNAs (tRNAs), and 1 non-coding region (GenBank accession number: MK617531). Four PCGs and two rRNAs are encoded on the light strand (*nad1*, *nad4*, *nad4L*, and *nad5*), while the other nine PCGs are located on the heavy strand.

The base composition was 36.0% A, 17.4% C, 12.1% G, and 34.5% T. The overall A + T content of the mitochondrial genome of *F. albicostatus* is 70.6%. Non-coding regions make up 438 bp, with the longest one speculated as the control region (264 bp), which is located between *srRNA* and *trnI*. The *srRNA* (AT content: 72.4%) and *lrRNA* (AT content: 74.6%) are 898 bp and 1302 bp, respectively. All PCGs have an ATD (ATA, ATT, or ATG) start codon, except *nad1*, which is initiated with GTG. Remarkably, *cox3*, *cob*, and *nad1-5* have incomplete stop codons (T- or TA-) and the remaining PCGs have the complete stop codon (TAA).

To investigate the phylogenetic position and the inner relationships of the suborder Balanomorph, phylogenetic trees were constructed with nucleotide sequences of 13 PCGs from 22 complete mitochondrial genomes of Sessilia, and *Lepas australis* (NC_025295) as outgroup species, using maximum likelihood with MEGA7 (Kumar et al. 2016) (Figure 1). In the tree, *F. albicostatus* is the most primitive member within the superfamily Balanoidea. Within the superfamily Balanoidea, the tree is divided into two parts. One part has two species of *Megabalanus* (*M. ajax* and *M. volcano*) cluster with *Acasta sulcata*, which belongs to Archaeobalanidae. Two are grouped with *Balauns balanus*, with being the most distantly related taxon. In another part, *Nobia grandis* and *Savignium* sp. BKKC-2013 are grouped together with high support (BP = 100), and then cluster with *Armatobalanus allium* and *Striatobalanus amaryllis*. The members of the Archaeobalanidae and Balanidae intermingle with species from Pyrgomatidae. The results supposed that Balanidae and Archaeobalanidae are non-monophyly, which is consistent with previous researches (Tsang et al. 2014, 2017; Shen et al. 2015).

**Figure 1.**
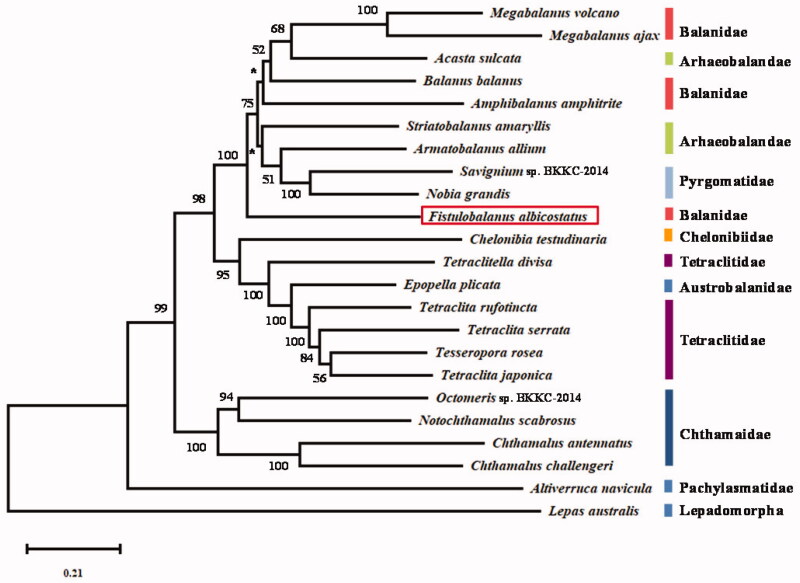
Maximum-likelihood phylogenetic tree based on 13 PCGs nucleotide acid sequences of *Fistulobalanus albicostatus* and 22 other Cirripedia species. Nodal supports are denoted on the corresponding branches for a bootstrap value >50% for ML, while *represents the value ≤50%.

The accession numbers of the genomes used for comparison are NC_024636 (*Megabalanus ajax*), NC_006293 (*Megabalanus volcano*), NC_029168 (*Acasta sulcata*), NC_024525 (*Amphibalanus amphitrite*), NC_029167 (*Armatobalanus allium*), KJ_754821 (*Savignium* sp. BKKC-2014), NC_023945 (*Nobia grandi*s), NC_029169 (*Chelonibia testudinaria*), NC_029170 (*Tetraclitella divisa*), NC_033393 (*Epopella plicata*), NC_037398 (*Tetraclita rufotincta*), NC_029154 (*Tetraclita serrata*), NC_037241 (*Tesseropora rosea*), NC_008974 (*Tetraclita japonica*), NC_026730 (*Chthamalus antennatus*), NC_022716 (*Notochthamalus scabrosus*), KJ_754820 (*Octomeris* sp. BKKC-2014), NC_005936 (*Pollicipes polymerus*), NC_025295 (*Lepas australi*s), and NC_026576 (*Lepas anserifera*).

## Data Availability

The data that support the findings of this study are openly available in GenBank of NCBI at https://www.ncbi.nlm.nih.gov, reference number MK617531.
